# The scientific value of numerical measures of human feelings

**DOI:** 10.1073/pnas.2210412119

**Published:** 2022-10-03

**Authors:** Caspar Kaiser, Andrew J. Oswald

**Affiliations:** ^a^Wellbeing Research Centre, University of Oxford, Oxford, OX1 3TD United Kingdom;; ^b^Institute for New Economic Thinking, Department of Social Policy and Intervention, University of Oxford, Oxford, OX1 2ER United Kingdom;; ^c^Department of Economics, CAGE Centre, University of Warwick, Coventry, CV4 7AL United Kingdom;; ^d^Institut zur Zukunft der Arbeit (IZA), Bonn, 53113 Germany

**Keywords:** happiness, pain, satisfaction, survey design, validity

## Abstract

Human feelings measured in integers (*my happiness is an 8 out of 10*, *my pain 2 out of 6*) have no objective scientific basis. They are “made-up” numbers on a scale that does not exist. Yet such data are extensively collected—despite criticism from, especially, economists—by governments and international organizations. We examine this paradox. We draw upon longitudinal information on the feelings and decisions of tens of thousands of randomly sampled citizens followed through time over four decades in three countries (*n* = 700,000 approximately). First, we show that a single feelings integer has greater predictive power than does a combined set of economic and social variables. Second, there is a clear inverse relationship between feelings integers and subsequent get-me-out-of-here actions (in the domain of neighborhoods, partners, jobs, and hospital visits). Third, this feelings-to-actions relationship takes a generic form, is consistently replicable, and is fairly close to linear in structure. Therefore, it seems that human beings can successfully operationalize an integer scale for feelings even though there is no true scale. How individuals are able to achieve this is not currently known. The implied scientific puzzle—an inherently cross-disciplinary one—demands attention.

## Significance

Human feelings cannot be expressed on a numerical scale. There are no units of measurement for feelings. However, such data are extensively collected in the modern world—by governments, corporations, and international organizations. Why? Our study finds that a feelings integer (like *my happiness is X out of 10*) has more predictive power than a collection of socioeconomic influences. Moreover, there is a clear link between those feelings numbers and later get-me-out-of-here actions. Finally, the feelings-to-actions relationship appears replicable and not too far from linear. Remarkably, therefore, humans somehow manage to choose their numerical answers in a systematic way as though they sense within themselves—and can communicate—a reliable numerical scale for their feelings. How remains an unsolved puzzle.

This paper studies the connection between feelings and actions. It uses cross-country longitudinal data (*n* = 700,000 approximately) to address a scientific enigma. The paper documents a foundational empirical pattern that has a consistent structure across diverse domains of life.

We begin with the following paradox. Today, large amounts of information are collected on feelings. Physicians ask patients to rate the level of their pain on a numerical scale (1, 2). The United Kingdom’s Office for National Statistics requires hundreds of thousands of citizens to answer life-satisfaction and happiness questions using a 0 to 10 scale (3). Multinational corporations email their customers to ask how satisfied they feel with purchases and quiz their own workers in job-satisfaction surveys. The world’s largest job-search site, Indeed.com, collects integers on employees’ happiness, scored out of 100, across thousands of workplaces.

Yet there is no empirical scale for feelings. Units of measurement do not exist for pain or happiness. Scientifically, a feelings integer is a “made-up” number. Critics argue that an analysis with such numbers is quasi-science and that integers for human feelings have no persuasive meaning.

Why, therefore, would organizations and governments want to collect these kinds of numerical scores? One possibility—as a scientific hypothesis—is that human beings are able to choose their integers in some well-founded, if currently poorly understood, way. Whether subconsciously or not, people may have a sense of an actual underlying scale for their innermost feelings, and their answers might trace out that scale. But could that be true? This paper offers evidence that suggests the answer is yes.

This paper is designed as a contribution to two types of scientific reader. The first, and principal, potential reader is a general scientist who is unfamiliar with, and skeptical of, the integers stated by humans in response to questions about their feelings. Initial disbelief in such numbers is appropriate for research scientists. For these readers, our aim is to provide what we hope is even-handed evidence on the power of made-up feelings integers.

The second type of potential reader is a researcher who wishes to use numerical information on feelings. For this latter group of readers (the literature includes refs. 4–16), who are less numerous in the broad scientific community and who already have to defend their work to nonbelieving colleagues, the intended contribution of the paper is a broader one. It is to offer a systematic testing method and uniform style of evidence base that is inspired by isolated findings in the early research literature. The paper’s unified approach uncovers a noticeable empirical consistency—visible in the paper’s later graphs.

The rationale for the paper’s testing method is that an individual’s internal feelings are intrinsically uncheckable in any direct way. People’s subsequent actions, however, can potentially unveil inner feelings. That is the idea upon which the paper builds.

Our analysis draws on longitudinal data from three nations. The objective was to assess whether there is evidence of a ubiquitous connection between *feelings integers* and what might be termed *get-me-out-of-here actions*. Such actions, explained more fully below, are where individuals choose to leave their current setting (in whatever domain of life). These are of special interest to scientific researchers because get-me-out-of-here actions can be taken to be unambiguous signals of latent human dissatisfaction with the prior status quo.

Scholars in many social science disciplines have discussed the analytical difficulties with feelings data (10, 17). Research has also shown i) that certain kinds of reported feelings are correlated with neurological and physiological outcomes (18–20). By its very nature, however, most of this work does not establish that feelings data are valid measures. In order to conclude, for example, that happiness can be observed in a certain part of the brain or bloodstream, it is necessary to begin by assuming that reported happiness numbers are meaningful. It is further known ii) that there is a statistical link between feelings data and much-later human outcomes, such as longevity, health, and future earnings (12, 18, 21); however, this literature is of a conceptually different kind, and although it valuably establishes that people’s answers are not random numbers, it cannot fulfill the corroborative objective of the current paper. Research has also found iii) that the intensity of smiling in the current period is correlated with well-being scores approximately 2 years later (22), iv) that observed expenditure shares on food are associated with shares predicted by life-satisfaction data (23), and that v) adjusted life-satisfaction rankings of US states are correlated with a ranking implied by a compensating-differentials model that uses objective data (24). Finally, it is known vi) that young doctors’ rankings of hospital residency locations and their reported levels of future anticipated happiness are correlated (6).

Critics believe that none of these studies yield general and definitive evidence on the believability of feelings integers. It is not possible to be sure that integers correspond reliably to internal mental states.

This may explain why the discipline of economics, for example, is still resistant to the use of numerical information on feelings (25–27). Other kinds of scientific investigators often take a different view. Psychology textbooks routinely draw upon data on human feelings. An example from the field of medicine is the presumption, as in refs. 1 and 2, that it is valuable to ask patients to rate their pain numerically. Another example from sociology is encapsulated in the so-called Thomas theorem (28), which states that if we define something as real or believe that something is real, it will genuinely be real in its consequences. Finally, some researchers suggest that feelings integers primarily reflect a frame of reference determined by social comparisons and past experiences (5, 29, 30).

Economists’ contrasting and long-standing mistrust may be due to a belief that data on human feelings carry little or no reliable predictive power. Milton Friedman—an early recipient of the Nobel Prize in Economics—suggested in a classic article on scientific methodology (31) that the ability to predict should be the fundamental benchmark for the assessment of scientific success. Consciously, we later follow Friedman’s strictures.

Inspired by early disparate findings about a potential link between subjective responses and behavior (32–37), we attempted to provide a unified framework to check and demonstrate the power of feelings integers. Different kinds of satisfaction data are observed at time t, and get-me-out-of-here behavior is observed at time t+1. We compared the performance of our models against a set of standard socioeconomic variables. The analysis used data from the United Kingdom, Germany, and Australia. It examined the relationship between get-me-out-of-here “exit” behavior and satisfaction in four domains: housing, intimate partnerships, jobs, and health.

## Results

How empirically informative are these kinds of made-up feelings numbers? The paper’s first calculation addresses this question. As shown in the righthand column of Table 1, feelings data here offer somewhat better predictive power (of subsequent observable actions) than do a combined set of standard economic and social variables. These economic and social variables include household income, relative income, employment status, establishment size, homeownership status, household size, number of children, marital status, education, and sets of region dummies. Of course, the extent to which these variables reflect the status quo varies. Income, for example, is an objective characteristic of jobs. Region dummies capture housing characteristics, and children are, in a sense, a characteristic of respondents’ marriage. However, we did not control for objective health variables; it was not possible to do that in a consistent way across countries and years.

**Table 1.** Get-me-out-of-here actions: Evidence of the greater predictive power of a single feelings integer compared with a combined set of standard economic and social variables

	Integer satisfaction*	Socioeconomic variables^†^	Which has greater predictive power?
Housing			
Germany	0.091	0.089	Integer satisfaction
United Kingdom	0.177	0.173	Integer satisfaction
Australia	0.195	0.220	Socioeconomic variables
Partner separation			
Germany	0.154	0.151	Integer satisfaction
United Kingdom	0.192	0.180	Integer satisfaction
Australia	0.193	0.191	Integer satisfaction
Job			
Germany	0.103	0.090	Integer satisfaction
United Kingdom	0.044	0.020	Integer satisfaction
Australia	0.200	0.186	Integer satisfaction
Hospitalization			
Germany	0.135	0.132	Integer satisfaction
United Kingdom	0.162	0.165	Socioeconomic variables
Australia	0.127	0.129	Socioeconomic variables

More predictive models are indicated in column 3. Satisfaction is measured each time in the year before the observed action (i.e., moving house, partner separation, changing job, moving to hospital). The datasets are longitudinal and cover 34 years of annual observations in Germany, 25 years in the United Kingdom, and 20 years in Australia. Note: The numbers in the columns are R-squared values. A larger adjusted R-squared, as indicated in green in the third column, implies higher predictive performance by a feelings integer. It can be seen that in 9 of 12 cases, a single value of integer satisfaction has higher explanatory power in predicting subsequent actions than does a set of economic and social variables. The regression equations that lie behind column 1 and column 2 include individual fixed-effects, age, age^2^, age^3^, and wave dummies. In column 1, integer satisfaction is entered linearly as a simple variable. In column 2, for the socioeconomic models, the equation uses a combined set of variables that are all included at the same time as independent variables: log household income, log relative income, self-employment, ln(#adults), ln(1+#children), marital status, childbirth, employment status, firm size, education, and state dummies. Satisfaction here is a scalar-valued variable. It is not entered as a set of satisfaction dummy variables (which, mechanically, would have even greater explanatory power). Data for the United Kingdom are taken from the UKHLS covering 1996 to 2020. German data are sourced from the SOEP between 1984 and 2018. Australian data are from the HILDA study, with years from 2001 to 2020. Samples sizes: Germany *n* = 274,618 (job), *n* = 279,509 (housing), *n* = 137,807 (partner separation), and *n* = 420,970 (hospitalization). United Kingdom *n* = 48,698 (job), *n* = 110,143 (housing), *n* = 74,115 (partner separation), and *n* = 222,384 (hospitalization). Australia *n* = 141,350 (job), *n* = 231,867 (housing), *n* = 139,738 (partner separation), and *n* = 47,041 (hospitalization).

^*^Entered as a single integer.

^†^Entered as a group of variables.

Full regression results are given in *SI Appendix*, Tables S5–S8.

In Table 1, we rely on a simple comparison of adjusted R-squared levels. However, the central finding is unchanged if we switch to other goodness-of-fit criteria (such as the mean squared error or “within” R-squared, as shown in *SI Appendix*, Table S3). Table 1 adjusts throughout for individual and survey wave fixed–effects, as well as for the age of the respondent.

It might be fair-minded to point out that in a few cases within Table 1 the feelings-integer predictive power is not substantially greater than that of the socioeconomic variables. A purist could also reasonably argue that 9 successes out of 12 is not a clinching statistical margin. Yet, that kind of reaction should perhaps be in seen in perspective. The key point is that a single made-up feelings number entered linearly in a regression performed fairly impressively when compared against a group of objective economic and social variables entered together in a regression. This even occurred when the included “objective” variables were a direct characteristic of the domain status quo in question (e.g., in the case of incomes in the domain of work).

Because there are institutional differences across nations—for example, in the way that housing and labor markets operate and also in divorce laws, we did not attempt comparisons of R-squared levels between one country and another.

It would be possible, of course, to combine feelings variables and socioeconomic variables within a regression equation. In a sense, that is what we did next.

**Fig. 1.**
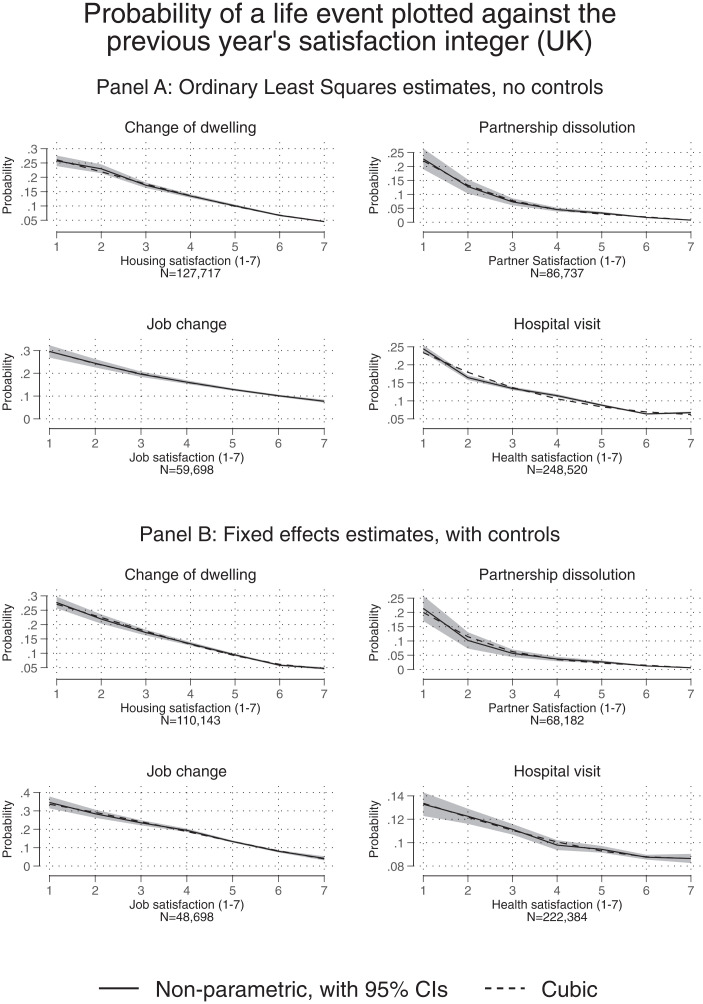
The predictive power of a feelings integer: United Kingdom. (*A*) Uncorrected correlations. (*B*) Fixed-effects regression-corrected correlations. *Notes:* These figures depict the relationship between a person’s chosen satisfaction integer in year *t* and a subsequent action in year *t*+1 by the person. A “cubic” specification here denotes a regression on a cubic in the integer value of satisfaction. A “nonparametric” specification denotes a regression on a full set of dummies for each stated level of satisfaction. Logit equations are given in the *SI Appendix*.

**Fig. 2.**
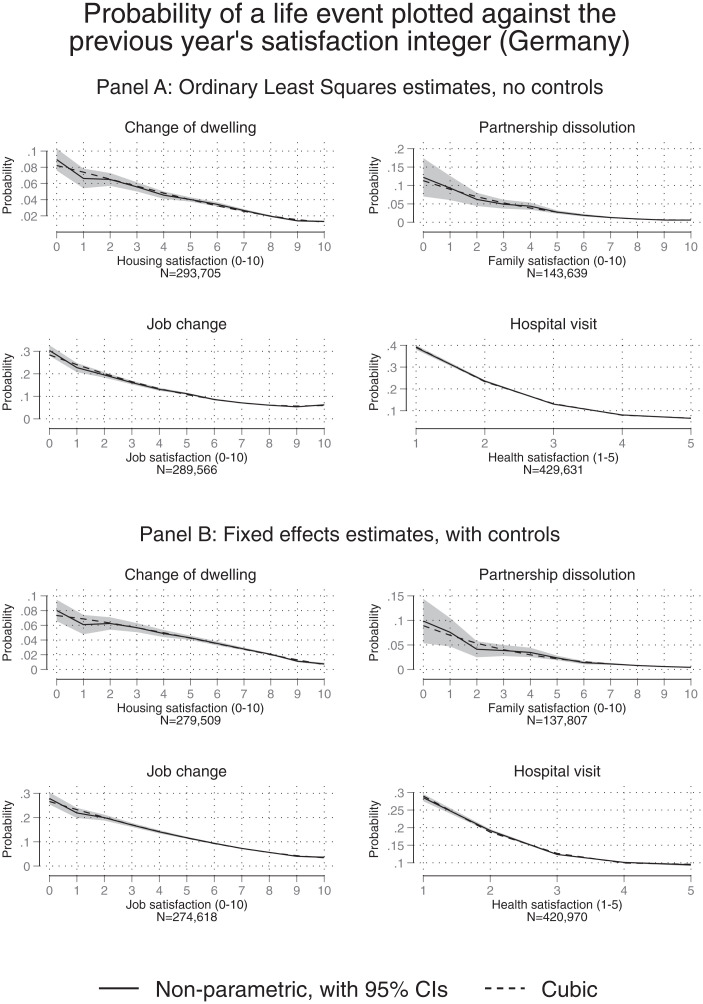
The predictive power of a feelings integer: Germany. (*A*) Uncorrected correlations. (*B*) Fixed-effects regression-corrected correlations. *Notes:* These figures depict the same kind of relationship between a person’s chosen satisfaction integer in year *t* and a subsequent action in year *t*+1 as also shown in Fig. 1. Here, results for Germany are shown. Logit equations are given in the *SI Appendix*.

**Fig. 3.**
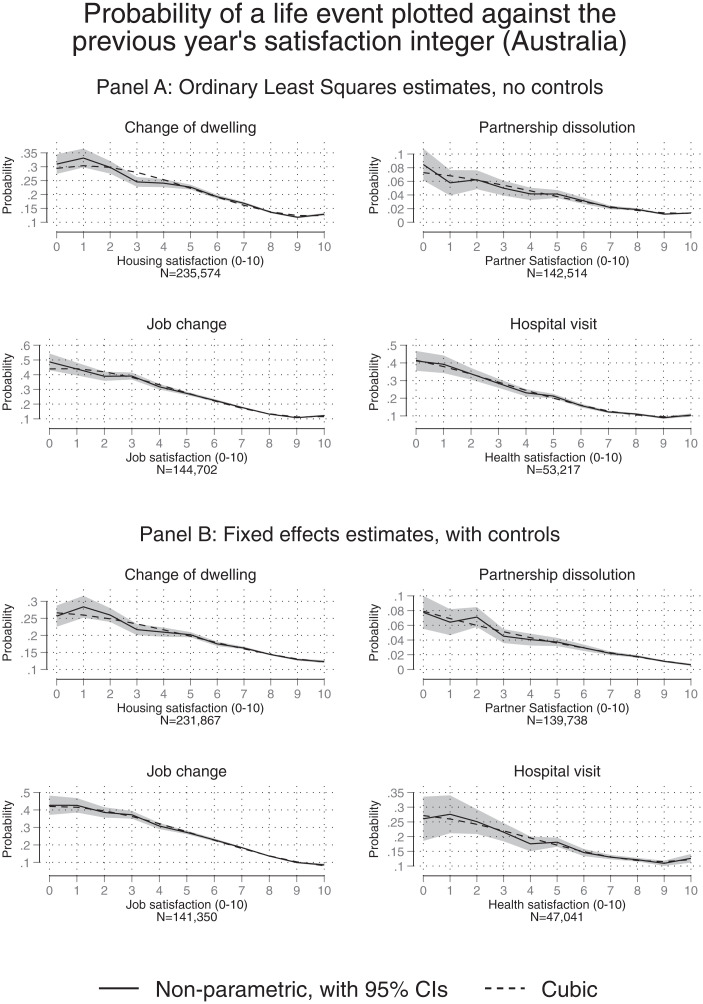
The predictive power of a feelings integer: Australia. (*A*) Uncorrected correlations. (*B*) Fixed-effects regression-corrected correlations. *Notes:* These figures depict the same kind of relationship between a person’s chosen satisfaction integer in year *t* and a subsequent action in year *t*+1 as also shown in Fig. 1. Here, results for Australia are shown. Logit equations are given in the *SI Appendix*.

The paper’s second substantive finding is illustrated in Figs. 1–3. For each country, the upper-half diagram, such as that given in Fig. 1*A*, depicts a correlation between a feelings integer and a subsequent observed action. The matching lower diagram (e.g., Fig. 1*B*) then gives the full regression-corrected pattern (these are within-person longitudinal findings). Evidence is repeated across countries, illustrating the relationships between integer feelings and actions in the United Kingdom, Germany, and Australia. For completeness, *SI Appendix*, Tables S9–S11 lay out the details of the underlying regression tables.

Each figure’s quadrant plots the relationship between a satisfaction level in year t and an outcome in year t+1. Patterns are presented here for four types of categories: moving dwellings, changing intimate partners, leaving jobs, and hospital visits. Of course, it would be sensible in the latter case to accept that the case of hospital visits is sometimes the result of a physician or family member taking a get-her/him-out-of-here decision. The graphs reveal a distinctive link between the integers that people state (about inner feelings) and what we have termed get-me-out-of-here actions.

For example, in the upper-left corner of Fig. 1 *A* and *B*, using data from the United Kingdom, the numbers 0.05…0.25 on the vertical axis correspond to the probabilities 5%…25% of a person moving away from his or her current dwelling. Satisfaction with housing measured in the previous period is treated as an integer on the horizontal axis. As shown in Fig. 1*A*, which shows the unadjusted correlation, the relationship is downward sloping, tightly defined statistically, and fairly close to linear in Probability/Satisfaction space (a formal check on the amount of divergence from linearity is presented in *SI Appendix*, Table S4). Even at this elementary level, therefore, it appears there is a great deal of systematic informational content in the integers offered by humans in surveys. These made-up numbers seem to have a notable degree of predictive ability.

Fig. 1*B*, in the lower half of the page, does the equivalent in a more complete way. The estimation is by fixed-effects equations, and a large number of independent variables (i.e., longitudinal covariates) are included in each regression equation. The outcome variables and independent variables are time varying. They are measured over large numbers of years, as detailed in the legends of the figures. Fig. 1*B* is within-person in a fully general sense: It adjusts for an individual’s changing year-on-year income, including marital circumstances, number of children, and other variables. These fixed-effects analyses therefore set a higher bar for statistical persuasiveness. In our context, the fixed-effects regressions are conditioning on each respondent’s exit probability across the entire observation period. Such conditioning implies that we estimate how deviations from a respondent’s average satisfaction level relate to deviations in a respondent’s average exit probability.

The pattern in Fig. 1*B* remains almost identical to that in Fig. 1*A*. This again suggests, as with the elementary approach of Fig. 1*A*, that feelings integers are here playing a reliable and substantial role as a predictor.

In the paper’s figures, some degree of generalizability is evident. For the United Kingdom, Fig. 1 *A* and *B* look noticeably similar across all life domain quadrants; that is, we see a very similar connection between feelings and actions in the domains of changing dwelling, change of partner, job change, and hospital visit. In each of these, an integer level of satisfaction is an inverse, monotonic, and tightly defined predictor of what will happen in the subsequent period. Except in the case of the quadrant for partnership dissolution, the relationships in these eight graphs look perhaps surprisingly close to linear (though not literally so).

Diagrammatic results are provided for two further nations. Fig. 2 *A* and *B* repeat the graphical procedure for the German data. Each time, the patterns that emerged were remarkably similar. Equivalent graphs for Australia are depicted in Fig. 3. Overall in these three countries, the integers chosen by randomly sampled individuals when asked about their feelings (with different aspects of life) are close to monotonic and relatively linear predictors of subsequent actions. A degree of generality applies across domains of life and for the three industrialized nations. It seems scientifically important eventually to know, and yet currently we do not, whether the observed pattern also holds in poorer, developing countries.

It should be emphasized that strict linearity of the function in Figs. 1–3, does not hold in our data. Instead, there is evidence in the figures of some degree of convexity.

How far do the statistical relationships diverge from being strictly linear functions? Not far, on balance, in our judgment. *SI Appendix*, Table S4 gives the adjusted R-squared values for several specifications that model the association between exit behavior and satisfaction. It can be seen that rather little additional explanatory power is gained when allowing for potential nonlinearities. This seems notable. It implies that, especially in the parts of the scale where most of the integer responses lie in the general population (i.e., between scores 7 and10 for most Australian and German data and scores 4 to 7 for UK data), the association between exit behavior and satisfaction is moderately well approximated by a linear equation.

## Discussion

This paper is a longitudinal study of feelings integers and get-me-out-of-here actions. It is written with two audiences in mind: general scientists who are (appropriately) skeptical of feelings integers and area specialists who have to work with and defend such data.

Feelings matter intensely to humans. Therefore, it seems natural and arguably essential for scientists to study feelings data. Although conventional economics has traditionally spurned such data, one strand in the general style of our argument has been discussed before by labor economists. They have demonstrated in cross-sectional estimations for the United States and United Kingdom that a low level of job satisfaction is associated with job separations (33, 34). More broadly, economists distinguish between “decision utility,” a representation of observable choice behavior, and “experienced utility,” which represents the unobserved quality of an individual’s feelings. Within the economist’s rational-agent framework, it is typically taken as axiomatic that decision utility and experienced utility coincide. In line with this, we showed that integer reports about experienced utility are—across multiple domains and countries—monotonically associated with the propensity for taking a get-me-out-of-here action. Therefore, integer feelings data and decision utility do seem to be tightly and consistently associated. However, we here cannot—and do not—argue that integer assessments are literally used in behavioral choices.

Our hope is that the paper’s findings will help in this area to bridge the current intellectual fissure between economics and psychology (and other disciplines). Using annual data on individuals from different nations over many decades, the paper illustrates the following points:

• In four domains of human life, a single feelings integer has more predictive power than a combined set of objective economic and social variables.
• There is an inverse relationship between these integers and subsequent get-me-out-of-here actions (in the domain of neighborhoods, intimate partners, jobs, and hospital visits).
• The relationship between feelings and actions is approximately monotonic and fairly close to linear.
• There is evidence—visible in the replicability across the paper’s graphs—of a previously undiscovered degree of consistency in feelings-action patterns across different domains.

In conclusion, human beings can apparently operationalize a reliable numerical scale for unmeasurable inner emotions. How they do this, using made-up integers on a scale that does not truly exist, is currently unknown. It demands further scientific investigation.

## Materials and Methods

We estimated regression-equation models of a given exit behavior on a domain-specific integer satisfaction variable. In each case, satisfaction variables were coded so that a larger integer indicates a higher level of satisfaction.

In the main, we concentrated on linear probability models estimated by ordinary least squares. Linear probability models have not only known disadvantages but also the important merit of allowing for the inclusion of individual fixed effects, which makes it possible to control for time-invariant, unobserved heterogeneity across respondents (e.g., unobserved familial background). However, Logit specifications as an alternative bivariate-outcome approach are reported in the *SI Appendix*. They produce the same conclusions.

Integer feelings variables are responses to simple questions such as *“How satisfied are you with your housing?”* where answers are recorded on an integer scale that here either ranged from 0 to 10 (in the German and Australian cases) or from 1 to 7 (in the UK case). In all cases, the variables were coded so that higher numbers indicated greater satisfaction. Response rates were high for these questions in our data. Across the three countries and the different well-being questions, the nonresponse rate averaged ∼2%. This seems to suggest that most respondents found it straightforward to translate their feelings/judgments (which have no natural units) into numbers.

Three nationally representative longitudinal datasets were used. The data are annual. Data for the United Kingdom were taken from the UK Household Longitudinal Study (UKHLS) covering 1996 to 2020. German data were sourced from the Socio-Economic Panel (SOEP) between 1984 and 2018. Australian data were from the Household, Income and Labour Dynamics (HILDA) study, with years from 2001 to 2020. Not all relevant variables were observed in every year. Our sample sizes ranged from 47,041 (Australia; Hospitalizations) to 429,631 (Germany; Hospitalizations). *SI Appendix*, Tables S1 and S2 provide descriptive material; they give the means and SDs of the key variables in our datasets.

The means on the get-me-out-of-here variables in *SI Appendix*, Table S1 are the observed rate of “exiting” in each of our four domains. In ∼10% of cases each year, respondents changed their job, stayed in a hospital, or changed their residence (in Germany, it should be noted, we could only observe movements of the household head, and that appears to be why the observed rate of changing residences is lower, at 2.4%, in the German data).

The observed annual partnership dissolution rate in *SI Appendix*, Table S1 is lower; it is ∼1–2% per year. Across the four domains, the survey respondents tended, on the numerical scale, to be most satisfied with their partners (in line with the comparatively low exit rate) and to be least satisfied with their health. The German and Australian surveys used the same 11-point scale for job, housing, and partner satisfaction. From these comparisons, we saw that in each of the domains, Australian citizens appeared to be more satisfied than German citizens. In the UK data, a 7-point scale was used.

Only those above age 18 years were included in our analysis. For specific domains, we imposed some additional sample restrictions: Concerning job quits, only those who were currently working or who had been working in the previous period were included. For partner separations, only those who were either currently married or in a partnership or who had been in a marriage/partnership in the previous period were included. For Germany, information on housing changes were only available for the household head. Because satisfaction data were not always available, the number of years covered slightly varied across domains. *SI Appendix*, Table S1 provides the exact coverage years.

Finally, in an appendix within the *SI Appendix*, we provide a brief calculus statement. The model there has a straightforward implication: Consistent with intuition, exit behavior at t+1 monotonically declined with *latent* (i.e., unobserved) satisfaction at t. Therefore, if exit behaviors were to monotonically decline in *reported* (i.e., observed) satisfaction integers, it would imply that respondents were indeed able to implement a numerical scale for their satisfaction. In this case, latent and reported feelings may approximately coincide in empirical practice.

## Supplementary Material

Supplementary File

## Data Availability

Some study data are available. Data for the United Kingdom are taken from the UKHLS covering 1996 to 2020. German data are sourced from the SOEP between 1984 and 2018. Australian data are from the HILDA study, with years from 2001 to 2020. These datasets are widely accessible for researchers, but we are not permitted to repost these datasets to a repository.
