# Supplementary material for: The scientific value of numerical measures of human feelings
Source: Proc Natl Acad Sci U S A. 2022 Oct 3;119(42):e2210412119. doi: 10.1073/pnas.2210412119 (PMC9586273; doi:10.1073/pnas.2210412119)

SUPPLEMENTARY FILE

Appendix A

Exploring why the probability of exit would decrease in respondents' satisfaction

Below is a simple derivation to illustrate why exit probabilities would be expected to decrease in true utility/satisfaction. A natural assumption is that reported satisfaction is an expression of latent satisfaction.

When considering whether to leave or to stay in a given environment (i.e. employer, partner, housing, health), individuals can be viewed as maximising the following:

$$V = pU + (1 - p)u - c(p) \tag{1}$$

Here, p is the probability of exiting the status quo, U is the actual satisfaction (or "utility") from the option that would obtain if an exit were to take place, and u is the level of actual satisfaction derived from the status quo. The term c represents a cost of searching for an outside option. We assume that, because it takes time and effort to search for longer in order to find a better option, the search cost c increases with p.

In effect, by choosing an outside-search intensity, individuals pick that p which maximizes V. Differentiating equation (1) with respect to p and setting the resulting expression to 0 yields the first-order condition:

$$V_p = U - u - c_p(p) = 0 \tag{2}$$

where subscripts indicate derivatives. Rearranging yields:

$$U - u = c_p \tag{3}$$

Differentiating with respect to u, we get:

$$-1/c_{pp} = dp/du \tag{4}$$

The second-order condition for a maximum requires:

$$-c_{pp} = V_{pp} < 0 \tag{5}$$

We therefore know that c_{pp} must be positive. Consequently, the gradient dp/du must be negative. Finally, in passing, it is worth noting that if the cost function c can be approximated to a second-order Taylor expansion, so that it is quadratic in p, its second derivative will be a constant. Equation (4) would, in that case, yield the prediction that the probability of exiting is linear in the status quo's satisfaction level.

Appendix B. Additional Tables

Table S1. Descriptive statistics for get-me-out-of-here exit variables

	N	Mean	SD (Min-Max)
		Housing	
Germany (1984-2018)	293,705	0.024	0.152 (0-1)
UK (1996-2009)	127,717	0.091	0.287 (0-1)
Australia (2001-2020)	235,574	0.150	0.357 (0-1)
		Partner separation	
Germany (2006-2018)	143,639	0.010	0.102 (0-1)
UK (1996-2009)	86,737	0.017	0.131 (0-1)
Australia (2001-2020)	142,514	0.018	0.134 (0-1)
		Job	
Germany (1984-2018)	289,566	0.080	0.271 (0-1)
UK (1996-2009)	59,698	0.132	0.339 (0-1)
Australia (2001-2020)	144,702	0.161	0.367 (0-1)
		Hospitalization	
Germany (1994-2018)	429,631	0.126	0.332 (0-1)
UK (1996-2008 & 2014-2020)	248,520	0.097	0.296 (0-1)
Australia (2003-2005, 2008-2010, 2012-2014, 2016-2018)	53,217	0.137	0.344 (0-1)

Note: In the German data, only the household head is observed as moving or not. For mechanical reasons, therefore, the observed rate of moving appears lower for Germany.

Table S2. Descriptive statistics for satisfaction variables (these are measured in the year before the get-me-out-of-here action)

	N	Mean	SD (Min-Max)
		Housing	
Germany	293,705	7.680	2.019 (0-10)
UK	127,717	5.436	1.433 (1-7)
Australia	235,574	7.876	1.722 (0-10)
		Partner separation	
Germany	143,639	8.282	1.605 (0-10)
UK	86,737	6.311	1.104 (1-7)
Australia	142,514	8.431	1.814 (0-10)
		Job	
Germany	289,566	7.177	1.995 (0-10)
UK	59,698	5.038	1.388 (1-7)
Australia	144,702	7.668	1.645 (0-10)
		Hospitalization	
Germany	429,631	3.402	0.953 (1-5)
UK	248,520	4.889	1.644 (1-7)
Australia	53,217	7.250	1.910 (0-10)

Note: Integer satisfaction data are coded in their rank-order, i.e. the first category is coded as ‘1’, the second category is coded as ‘2’, etc.

Table S3. Mean squared error and within R-squared for models of exit behaviour using either integer satisfaction data (column 1) or standard socio-economic variables (column 2). More predictive models are indicated in column 3.

	Integer Satisfaction (entered as a single integer)	*Socio-Economic Variables (entered as a group of variables)*	*Which Has Greater Predictive Power?*
	(1)	*(2)*	*(3)*
		Housing	
Germany	0.1335 (0.0124)	0.1336 (0.0110)	Integer Satisfaction
UK	0.2346 (0.0304)	0.2353 (0.0257)	Integer Satisfaction
Australia	0.3034 (0.0139)	0.2986 (0.0452)	Socio-economic Variables
		Partner separation	
Germany	0.0793 (0.0090)	0.0794 (0.0058)	Integer Satisfaction
UK	0.0723 (0.0161)	0.0727 (0.0029)	Integer Satisfaction
Australia	0.1038 (0.0114)	0.1039 (0.0094)	Integer Satisfaction
		Job	
Germany	0.2656 (0.0213)	0.2670 (0.0078)	Integer Satisfaction
UK	0.3015 (0.0327)	0.3050 (0.0096)	Integer Satisfaction
Australia	0.3058 (0.0387)	0.3085 (0.0216)	Integer Satisfaction
		Hospitalization	
Germany	0.2909 (0.0110)	0.2915 (0.0071)	Integer Satisfaction
UK	0.2423 (0.0048)	0.2419 (0.0088)	Socio-economic Variables
Australia	0.2634 (0.0100)	0.2630 (0.0129)	Socio-economic Variables

Note: The first number in each column are mean squared errors (MSE). A smaller MSE, as indicated in green, implies better predictive performance. The MSE is given by $N^{-1}\sum_i(\hat{y}_i - y_i)^2$, where N denotes the sample size, y_i is a dummy for observed exit behaviour, and $\hat{y}_i$ denotes the predicted probability of engaging in an exit behaviour. The numbers in parentheses are within-R-squared values. A larger within R-squared implies higher predictive performance.

Both types of models include individual fixed-effects, age, age^2, age^3, and wave dummies. Integer satisfaction is entered linearly as a single variable. Socio-economic models include log household income and relative income, self-employment, ln(#adults), ln(1+#children), marital status, childbirth, employment status, firm size, education, state dummies.

Samples sizes: Germany N=271,684 (job), N=279,509 (housing), N=137,807 (partner separation), N=420,970 (hospitalization). UK N=48,698 (job), N=110,143 (housing), N=74,115 (partner separation), N=222,384 (hospitalization). Australia N=141,350 (job), N=231,867 (housing), N=139,738 (partner separation), N=47,041 (hospitalization).

Table S4. Evidence of Minimal Divergence from Linearity of the Relationship Between Feelings Integers and Get-Me-Out-Of-Here Actions (Feelings Integers are Measured in Year t and Get-Me-Out-Of-Here Actions in Year t+1)

Adjusted R-squared Levels for Linear and Non-linear Specifications in the Actions Equation

	Neighbourhood satisfaction	Family / Partner satisfaction	Job satisfaction	Health satisfaction
		UK		
**Linear**	**0.1897**	**0.1932**	**0.0450**	**0.1657**
Cubic	0.1906	0.1985	0.0450	0.1658
Non-parametric	0.1907	0.1989	0.0450	0.1658
		Germany		
**Linear**	**0.0945**	**0.1544**	**0.1050**	**0.1371**
Cubic	0.0945	0.1553	0.1057	0.1392
Non-parametric	0.0946	0.1554	0.1057	0.1393
		Australia		
**Linear**	**0.2231**	**0.1954**	**0.2065**	**0.1319**
Cubic	0.2232	0.1954	0.2069	0.1325
Non-parametric	0.2232	0.1955	0.2070	0.1326

Note: **"**Adjusted R-squared" is the adjusted R-squared from the regressions underlying Figures 1b, 2b, 3b, and from equivalent regressions in which satisfaction is entered linearly. The mildness of the divergence from linearity can be seen by comparing, for example, 0.1897 to 0.1907 in the top left-hand corner.

Table S5. Regressions of housing exits on a satisfaction integer (odd columns) or a set of standard economic and social variables (even columns)

	(1) UK, satisfaction	(2) UK, socio-economic variables	(3) Germany, satisfaction	(4) Germany, socio-economic variables	(5) Australia, satisfaction	(6) Australia, socio-economic variables
Housing/Neighbourhood satisfaction integer	-0.038^{***}		-0.007^{***}		-0.018^{***}	
	(0.001)		(0.000)		(0.001)	
ln(HH income)		-0.004		0.008^{***}		-0.000
		(0.003)		(0.001)		(0.001)
ln(ref income)		0.008		0.005		0.052^{***}
		(0.017)		(0.006)		(0.013)
ln(job hours)		-0.004^{+}		-0.001^{*}		0.006^{*}
		(0.002)		(0.000)		(0.002)
ln(# adults in HH)		0.045^{***}		0.035^{***}		0.014^{***}
		(0.006)		(0.002)		(0.003)
ln(# children in HH)		-0.034^{***}		0.008^{***}		-0.036^{***}
		(0.005)		(0.002)		(0.003)
Child birth		0.027^{***}		-0.003		0.001
		(0.006)		(0.002)		(0.005)
Self-employed		-0.015^{+}		-0.003		-0.009^{*}
		(0.008)		(0.002)		(0.004)
Stable relationship (but not married)		0.009				0.014^{**}
		(0.008)				(0.005)
Separated		0.030^{***}		0.021^{***}		0.050^{***}
		(0.008)		(0.002)		(0.008)
Divorced		0.050^{***}		0.011^{***}		0.020^{*}
		(0.011)		(0.003)		(0.008)
Widowed		0.065^{***}		0.015^{***}		0.027^{***}
		(0.013)		(0.004)		(0.007)
Never married		0.090^{***}		0.031^{***}		0.070^{***}
		(0.012)		(0.004)		(0.007)
Other marital status				0.007		
				(0.009)		
Unemployed		-0.005		-0.006^{+}		0.039^{***}
		(0.009)		(0.003)		(0.010)
Other non-working		-0.009		-0.007^{*}		0.023^{**}
		(0.007)		(0.003)		(0.008)
Retired		-0.013^{*}		-0.009^{**}		
		(0.007)		(0.003)		
Small firm		0.001		-0.005		
		(0.008)		(0.003)		
Large firm		-0.003		-0.005^{+}		-0.001
		(0.009)		(0.003)		(0.003)
Tertiary education		0.024^{+}		-0.013^{*}		0.045^{***}
		(0.013)		(0.005)		(0.008)
Home owner		-0.153^{***}		-0.012^{***}		-0.213^{***}
		(0.008)		(0.002)		(0.004)
Age	-0.031^{***}	-0.012^{*}	-0.018^{***}	-0.016^{***}	0.012^{**}	0.013^{**}
	(0.005)	(0.005)	(0.001)	(0.001)	(0.004)	(0.004)
Age-squared	0.000^{***}	0.000	0.000^{***}	0.000^{***}	0.000^{**}	0.000
	(0.000)	(0.000)	(0.000)	(0.000)	(0.000)	(0.000)
Age-cubed	-0.000^{***}	0.000	-0.000^{***}	-0.000^{***}	0.000	0.000^{*}
	(0.000)	(0.000)	(0.000)	(0.000)	(0.000)	(0.000)
Constant	0.980^{***}	0.661^{**}	0.434^{***}	0.202^{***}	-0.264^{+}	-0.765^{***}
	(0.155)	(0.219)	(0.018)	(0.053)	(0.146)	(0.190)
Observations	110143	110143	279509	279509	231867	231867
Adjusted R^2	0.177	0.173	0.091	0.089	0.195	0.220

Note: Clustered standard errors in parentheses. + $p < 0.1$ * $p < 0.05$, ** $p < 0.01$, *** $p < 0.001$. Wave fixed-

effects are included in all regressions. Region fixed-effects are only included in the socio-economic models.

Table S6. Regressions of partner exits on a satisfaction integer (odd columns) or a set of standard economic and social variables (even columns)

	(1) UK, satisfaction	(2) UK, socio-economic variables	(3) Germany, satisfaction	(4) Germany, socio-economic variables	(5) Australia, satisfaction	(6) Australia, socio-economic variables
Family/Partner satisfaction integer	-0.016***		-0.005***		-0.006***	
	(0.001)		(0.000)		(0.000)	
ln(HH income)		-0.001		-0.000		0.000
		(0.002)		(0.001)		(0.000)
ln(ref income)		0.012		0.001		0.020*
		(0.009)		(0.009)		(0.008)
ln(job hours)		-0.001		0.001**		0.000
		(0.001)		(0.000)		(0.001)
ln(# adults in HH)		-0.003		0.004+		0.004
		(0.005)		(0.003)		(0.003)
ln(# children in HH)		-0.001		-0.004*		0.006***
		(0.003)		(0.002)		(0.002)
Child birth		-0.005+		-0.004*		-0.005**
		(0.003)		(0.002)		(0.002)
Self-employed		-0.005		-0.001		0.000
		(0.004)		(0.003)		(0.002)
Stable relationship (but not married)		-0.002				0.030***
		(0.004)				(0.002)
Unemployed		-0.001		-0.000		0.003
		(0.006)		(0.003)		(0.005)
Other non-working		-0.002		0.000		0.001
		(0.004)		(0.002)		(0.003)
Retired		-0.002		0.001		
		(0.003)		(0.003)		
Small firm		0.006		-0.001		
		(0.004)		(0.002)		
Large firm		0.003		-0.000		-0.003*
		(0.004)		(0.002)		(0.001)
Tertiary education		-0.011		0.001		-0.019**
		(0.009)		(0.009)		(0.006)
Home owner		-0.002		-0.002		-0.004+
		(0.004)		(0.002)		(0.002)
Age	0.001	0.003	0.017***	0.018***	-0.006**	-0.003
	(0.003)	(0.004)	(0.002)	(0.002)	(0.002)	(0.003)
Age-squared	-0.000	-0.000	-0.000***	-0.000***	0.000	-0.000
	(0.000)	(0.000)	(0.000)	(0.000)	(0.000)	(0.000)
Age-cubed	0.000	0.000	0.000***	0.000***	0.000	0.000*
	(0.000)	(0.000)	(0.000)	(0.000)	(0.000)	(0.000)
Constant	0.085	-0.120	-0.333***	-0.417***	0.225**	-0.107
	(0.081)	(0.120)	(0.036)	(0.087)	(0.076)	(0.115)
Observations	74115	74115	137807	137807	139738	139738
Adjusted R^2	0.192	0.180	0.154	0.151	0.193	0.191

Note: Clustered standard errors in parentheses. + p < 0.1 * p < 0.05, ** p < 0.01, *** p < 0.001. Wave fixed-effects are included in all regressions. Region fixed-effects are only included in the socio-economic models.

Table S7. Regressions of job exits on a satisfaction integer (odd columns) or a set of standard economic and social variables (even columns)

	(1)<br>UK, satisfaction	(2)<br>UK, socio-economic variables	(3)<br>Germany, satisfaction	(4)<br>Germany, socio-economic variables	(5)<br>Australia, satisfaction	(6)<br>Australia, socio-economic variables
Job satisfaction integer	-0.051***		-0.021***		-0.040***	
	(0.002)		(0.000)		(0.001)	
ln(HH income)		0.032***		-0.009***		-0.004**
		(0.007)		(0.003)		(0.001)
ln(ref income)		0.017		-0.060***		-0.036
		(0.034)		(0.012)		(0.023)
ln(job hours)		-0.004		-0.001		-0.068***
		(0.005)		(0.001)		(0.004)
ln(# adults in HH)		0.010		0.007**		0.006
		(0.009)		(0.003)		(0.004)
ln(# children in HH)		0.003		-0.002		-0.016***
		(0.008)		(0.002)		(0.004)
Child birth		0.011		-0.007*		0.000
		(0.011)		(0.003)		(0.006)
Self-employed		-0.066**		-0.078***		-0.064***
		(0.020)		(0.005)		(0.006)
Stable relationship (but not married)		0.007				0.009+
		(0.010)				(0.006)
Separated		0.011		0.006		0.001
		(0.027)		(0.009)		(0.009)
Divorced		0.042*		0.004		-0.001
		(0.016)		(0.004)		(0.009)
Widowed		0.022		0.024***		0.020
		(0.017)		(0.005)		(0.018)
Never married		0.003		0.010*		0.033***
		(0.015)		(0.004)		(0.008)
Other marital status				-0.007		
				(0.013)		
Small firm		0.094***		0.016***		
		(0.024)		(0.004)		
Large firm		0.097***		-0.002		-0.037***
		(0.024)		(0.004)		(0.003)
Tertiary education		0.012		0.011+		0.001
		(0.019)		(0.006)		(0.012)
Home owner		0.017+		-0.002		-0.002
		(0.010)		(0.002)		(0.004)
Age	-0.028**	-0.034**	-0.009***	-0.007**	-0.044***	-0.030***
	(0.010)	(0.011)	(0.002)	(0.002)	(0.006)	(0.006)
Age-squared	-0.000	-0.000	0.000	0.000	0.001***	0.001***
	(0.000)	(0.000)	(0.000)	(0.000)	(0.000)	(0.000)
Age-cubed	0.000+	0.000	0.000*	0.000+	-0.000***	-0.000***
	(0.000)	(0.000)	(0.000)	(0.000)	(0.000)	(0.000)
Constant	1.459***	0.737+	0.502***	0.920***	1.108***	1.228***
	(0.260)	(0.418)	(0.028)	(0.126)	(0.161)	(0.294)
Observations	48698	48698	274618	274618	141350	141350
Adjusted R^2	0.044	0.020	0.103	0.090	0.200	0.186

Note: Clustered standard errors in parentheses. + $p < 0.1$ * $p < 0.05$, ** $p < 0.01$, *** $p < 0.001$. Wave fixed-effects are included in all regressions. Region fixed-effects are only included in the socio-economic models.

Table S8. Regressions of hospitalisations on a satisfaction integer (odd columns) or a set of standard economic and social variables (even columns).

	(1) UK, satisfaction	(2) UK, soc-economic variables	(3) Germany, satisfaction	(4) Germany, soc-economic variables	(5) Australia, satisfaction	(6) Australia, soc-economic variables
Health satisfaction integer	-0.008***		-0.040***		-0.015***	
	(0.001)		(0.001)		(0.002)	
ln(HH income)		0.003		0.005*		0.002
		(0.002)		(0.002)		(0.003)
ln(ref income)		0.007		-0.016		-0.039
		(0.013)		(0.011)		(0.031)
ln(job hours)		0.003*		0.004***		-0.017**
		(0.002)		(0.000)		(0.005)
ln(# adults in HH)		-0.021***		-0.011***		-0.004
		(0.004)		(0.003)		(0.006)
ln(# children in HH)		-0.066***		-0.048***		-0.064***
		(0.003)		(0.003)		(0.006)
Child birth		-0.055***		0.015***		0.004
		(0.005)		(0.003)		(0.010)
Self-employed		0.003		-0.007+		0.002
		(0.005)		(0.004)		(0.009)
Stable relationship (but not married)		-0.028***				-0.061***
		(0.005)				(0.009)
Separated		-0.002		0.003		-0.057***
		(0.009)		(0.008)		(0.015)
Divorced		-0.033***		-0.002		-0.069***
		(0.007)		(0.005)		(0.015)
Widowed		-0.040***		-0.017**		-0.016
		(0.008)		(0.006)		(0.020)
Never married		-0.071***		-0.056***		-0.099***
		(0.006)		(0.004)		(0.012)
Other marital status				0.004		
				(0.016)		
Unemployed		0.002		0.019***		-0.072***
		(0.006)		(0.005)		(0.022)
Other non-working		0.007		0.028***		-0.041*
		(0.005)		(0.004)		(0.019)
Retired		0.008		0.038***		
		(0.006)		(0.005)		
Small firm		-0.007		0.001		
		(0.005)		(0.004)		
Large firm		0.000		0.003		0.008
		(0.006)		(0.004)		(0.006)
Tertiary education		-0.000		0.028***		0.030*
		(0.008)		(0.005)		(0.014)
Home owner		0.014***		-0.002		0.002
		(0.004)		(0.003)		(0.007)
Age	-0.003	0.000	-0.006***	-0.007***	0.011	0.014
	(0.003)	(0.004)	(0.001)	(0.002)	(0.009)	(0.010)
Age-squared	-0.000	-0.000***	0.000	0.000	-0.000+	-0.000*
	(0.000)	(0.000)	(0.000)	(0.000)	(0.000)	(0.000)
Age-cubed	0.000***	0.000***	0.000**	0.000**	0.000***	0.000***
	(0.000)	(0.000)	(0.000)	(0.000)	(0.000)	(0.000)
Constant	0.231*	0.139	0.355***	0.399***	-0.135	0.205
	(0.106)	(0.162)	(0.022)	(0.109)	(0.358)	(0.476)
Observations	222384	222384	420970	420970	47041	47041
Adjusted R^2	0.162	0.165	0.135	0.132	0.127	0.129

Note: Clustered standard errors in parentheses. + p < 0.1 * p < 0.05, ** p < 0.01, *** p < 0.001. Wave fixed-

effects are included in all regressions. Region fixed-effects are only included in the socio-economic models.

Table S9. Full fixed-effects regressions of exits in the domains of intimate partners, housing, jobs, and health on satisfaction integers and a set of controls (UK). This table corresponds to the non-parametric results in Figure 1b and the non-parametric results for the UK in Table 2.

	(1)<br>Housing exit	(2)<br>Intimate partner exit	(3)<br>Job exit	(4)<br>Hospitalisation
Satisfaction integer=2	-0.058***	-0.112***	-0.061**	-0.010+
	(0.012)	(0.027)	(0.020)	(0.006)
Satisfaction integer=3	-0.105***	-0.157***	-0.111***	-0.021***
	(0.011)	(0.023)	(0.018)	(0.006)
Satisfaction integer=4	-0.144***	-0.177***	-0.151***	-0.035***
	(0.011)	(0.023)	(0.017)	(0.006)
Satisfaction integer=5	-0.183***	-0.187***	-0.212***	-0.039***
	(0.011)	(0.023)	(0.017)	(0.006)
Satisfaction integer=6	-0.219***	-0.201***	-0.265***	-0.045***
	(0.011)	(0.023)	(0.017)	(0.006)
Satisfaction integer=7	-0.230***	-0.208***	-0.304***	-0.046***
	(0.011)	(0.023)	(0.018)	(0.006)
ln(HH income)	-0.003	-0.000	0.029***	0.003
	(0.003)	(0.002)	(0.007)	(0.002)
ln(ref income)	-0.003	0.013	0.007	0.007
	(0.017)	(0.009)	(0.033)	(0.013)
ln(job hours)	-0.004+	-0.002	-0.004	0.003*
	(0.002)	(0.001)	(0.004)	(0.002)
ln(# adults in HH)	0.041***	-0.005	0.010	-0.022***
	(0.006)	(0.004)	(0.009)	(0.004)
ln(# children in HH)	-0.038***	-0.002	0.003	-0.065***
	(0.005)	(0.003)	(0.008)	(0.003)
Child birth	0.027***	-0.004	0.011	-0.055***
	(0.006)	(0.003)	(0.011)	(0.005)
Self-employed	-0.016*	-0.006	-0.066***	0.003
	(0.008)	(0.004)	(0.020)	(0.005)
Stable relationship	0.010	-0.001	0.008	-0.028***
(but not married)	(0.007)	(0.004)	(0.010)	(0.005)
Separated	0.028***		0.003	-0.003
	(0.008)		(0.028)	(0.009)
Divorced	0.045***		0.036*	-0.034***
	(0.011)		(0.017)	(0.007)
Widowed	0.059***		0.023	-0.040***
	(0.013)		(0.017)	(0.008)
Never married	0.095***		0.005	-0.071***
	(0.011)		(0.014)	(0.006)
Unemployed	-0.007	-0.003		0.002
	(0.009)	(0.006)		(0.006)
Other non-working	-0.008	-0.002		0.005
	(0.007)	(0.004)		(0.005)
Retired	-0.011	-0.001		0.009
	(0.007)	(0.003)		(0.005)
Small firm	0.002	0.007+	0.093***	-0.007
	(0.008)	(0.004)	(0.024)	(0.005)
Large firm	-0.002	0.004	0.095***	0.001
	(0.009)	(0.004)	(0.024)	(0.006)
Tertiary education	0.026+	-0.009	0.017	-0.000
	(0.013)	(0.009)	(0.018)	(0.008)
Home owner	-0.135***	-0.001	0.015	0.014***
	(0.008)	(0.005)	(0.009)	(0.004)
Age-squared	-0.000	-0.000	-0.001***	-0.000***
	(0.000)	(0.000)	(0.000)	(0.000)
Age-cubed	0.000**	0.000	0.000***	0.000***
	(0.000)	(0.000)	(0.000)	(0.000)

Constant	0.506**	0.147	0.372	0.188
	(0.187)	(0.117)	(0.344)	(0.136)
Observations	110143	68182	48698	222384
Adjusted R^2	0.191	0.199	0.045	0.166

Note: Clustered standard errors in parentheses. + p < 0.1 * p < 0.05, ** p < 0.01, *** p < 0.001. Wave and region fixed-effects are included in all regressions. Satisfaction integer=0 is omitted and acts as the reference category.

Table S10. Full fixed-effects regressions of exits in the domains of intimate partners, housing, jobs, and health on satisfaction integers and a set of controls (Germany). This table corresponds to the non-parametric results in Figure 2b and the non-parametric results for Germany in Table 2.

	(1)<br>Housing exit	(2)<br>Intimate partner exit	(3)<br>Job exit	(4)<br>Hospitalisation
Satisfaction integer=1	-0.019*	-0.023	-0.060***	-0.094***
	(0.010)	(0.027)	(0.016)	(0.005)
Satisfaction integer=2	-0.018*	-0.058*	-0.079***	-0.161***
	(0.008)	(0.024)	(0.014)	(0.005)
Satisfaction integer=3	-0.023**	-0.060*	-0.109***	-0.184***
	(0.008)	(0.024)	(0.013)	(0.006)
Satisfaction integer=4	-0.031***	-0.064**	-0.138***	-0.191***
	(0.008)	(0.024)	(0.013)	(0.006)
Satisfaction integer=5	-0.037***	-0.075**	-0.162***	
	(0.008)	(0.023)	(0.013)	
Satisfaction integer=6	-0.045***	-0.085***	-0.185***	
	(0.008)	(0.023)	(0.013)	
Satisfaction integer=7	-0.052***	-0.087***	-0.206***	
	(0.007)	(0.023)	(0.013)	
Satisfaction integer=8	-0.060***	-0.091***	-0.223***	
	(0.007)	(0.023)	(0.013)	
Satisfaction integer=9	-0.069***	-0.093***	-0.239***	
	(0.007)	(0.023)	(0.013)	
Satisfaction integer=10	-0.073***	-0.094***	-0.243***	
	(0.007)	(0.023)	(0.013)	
ln(HH income)	0.009***	-0.000	-0.007**	0.007***
	(0.001)	(0.001)	(0.003)	(0.002)
ln(ref income)	0.007	-0.000	-0.057***	-0.019+
	(0.006)	(0.009)	(0.012)	(0.010)
ln(job hours)	-0.001*	0.000**	-0.001	0.004***
	(0.000)	(0.000)	(0.001)	(0.000)
ln(# adults in HH)	0.033***	0.003	0.008**	-0.012***
	(0.002)	(0.003)	(0.003)	(0.003)
ln(# children in HH)	0.007***	-0.005*	-0.001	-0.046***
	(0.002)	(0.002)	(0.002)	(0.003)
Child birth	-0.003	-0.003+	-0.007*	0.018***
	(0.002)	(0.002)	(0.003)	(0.003)
Self-employed	-0.003	-0.001	-0.074***	-0.006
	(0.002)	(0.003)	(0.005)	(0.004)
Single	0.030***		0.009*	-0.056***
	(0.004)		(0.004)	(0.004)
Widowed	0.021***		0.010	0.001
	(0.002)		(0.009)	(0.007)
Divorced	0.011***		0.005	-0.001
	(0.003)		(0.004)	(0.005)
Separated	0.014***		0.026***	-0.017**
	(0.004)		(0.005)	(0.005)
Other	0.006		-0.006	0.002
	(0.009)		(0.012)	(0.016)
Unemployed	-0.006+	-0.000		0.012**
	(0.003)	(0.003)		(0.004)
Other non-working	-0.007*	0.001		0.027***
	(0.003)	(0.002)		(0.004)
Retired	-0.009**	0.001		0.040***
	(0.003)	(0.003)		(0.005)
Small firm	-0.005	-0.001	0.017***	0.001
	(0.003)	(0.002)	(0.004)	(0.004)
Large firm	-0.005+	-0.000	0.001	0.002
	(0.003)	(0.002)	(0.004)	(0.004)

Tertiary education	-0.013*	0.002	0.012^{+}	0.030***
	(0.005)	(0.009)	(0.006)	(0.005)
Home owner	-0.005*	-0.002	-0.002	-0.001
	(0.002)	(0.002)	(0.002)	(0.002)
Age-squared	0.000***	-0.000***	-0.000	0.000^{+}
	(0.000)	(0.000)	(0.000)	(0.000)
Age-cubed	-0.000***	0.000***	0.000*	0.000^{+}
	(0.000)	(0.000)	(0.000)	(0.000)
Constant	-0.256***	0.537***	0.944***	0.304**
	(0.061)	(0.136)	(0.112)	(0.102)
Observations	279509	137807	274618	420970
Adjusted R^2	0.095	0.155	0.106	0.139

Note: Clustered standard errors in parentheses. + p < 0.1 * p < 0.05, ** p < 0.01, *** p < 0.001. Wave and region fixed-effects are included in all regressions. Satisfaction integer=0 is omitted and acts as the reference category.

Table S11. Full fixed-effects regressions of exits in the domains of intimate partners, housing, jobs, and health on satisfaction integers and a set of controls (Australia). This table corresponds to the non-parametric results in Figure 2b and the non-parametric results for Germany in Table 2.

	(1)<br>Housing exit	(2)<br>Intimate partner exit	(3)<br>Job exit	(4)<br>Hospitalisation
Satisfaction integer=1	0.027	-0.013	-0.000	0.015
	(0.021)	(0.014)	(0.035)	(0.048)
Satisfaction integer=2	0.003	-0.006	-0.041	-0.010
	(0.018)	(0.013)	(0.032)	(0.041)
Satisfaction integer=3	-0.040*	-0.032**	-0.055+	-0.045
	(0.017)	(0.012)	(0.030)	(0.040)
Satisfaction integer=4	-0.047**	-0.037**	-0.119***	-0.085*
	(0.017)	(0.012)	(0.029)	(0.040)
Satisfaction integer=5	-0.054***	-0.040***	-0.155***	-0.079*
	(0.016)	(0.012)	(0.029)	(0.039)
Satisfaction integer=6	-0.081***	-0.048***	-0.198***	-0.115**
	(0.016)	(0.012)	(0.028)	(0.039)
Satisfaction integer=7	-0.094***	-0.056***	-0.242***	-0.130***
	(0.016)	(0.012)	(0.028)	(0.039)
Satisfaction integer=8	-0.113***	-0.060***	-0.291***	-0.139***
	(0.016)	(0.012)	(0.028)	(0.039)
Satisfaction integer=9	-0.128***	-0.067***	-0.328***	-0.151***
	(0.016)	(0.011)	(0.028)	(0.039)
Satisfaction integer=10	-0.134***	-0.071***	-0.341***	-0.134***
	(0.016)	(0.011)	(0.028)	(0.040)
ln(HH income)	-0.000	0.000	-0.002	0.003
	(0.001)	(0.000)	(0.001)	(0.003)
ln(ref income)	0.050***	0.020*	-0.029	-0.044
	(0.013)	(0.008)	(0.023)	(0.031)
ln(job hours)	0.006**	0.000	-0.068***	-0.014**
	(0.002)	(0.001)	(0.003)	(0.005)
ln(# adults in HH)	0.013***	0.001	0.007	-0.006
	(0.003)	(0.003)	(0.004)	(0.006)
ln(# children in HH)	-0.034***	0.004*	-0.016***	-0.064***
	(0.003)	(0.002)	(0.004)	(0.006)
Child birth	0.000	-0.004*	0.000	0.005
	(0.005)	(0.002)	(0.006)	(0.010)
Self-employed	-0.008*	0.000	-0.059***	0.003
	(0.004)	(0.002)	(0.006)	(0.009)
Stable relationship	0.013**	0.031***	0.008	-0.061***
(but not married)	(0.005)	(0.002)	(0.006)	(0.009)
Separated	0.049***		0.003	-0.058***
	(0.008)		(0.009)	(0.015)
Divorced	0.019*		-0.001	-0.072***
	(0.008)		(0.009)	(0.015)
Widowed	0.027***		0.025	-0.017
	(0.007)		(0.017)	(0.020)
Never married	0.067***		0.036***	-0.103***
	(0.007)		(0.008)	(0.012)
Unemployed	0.039***	-0.001		-0.055**
	(0.010)	(0.005)		(0.021)
Other non-working	0.023**	-0.001		-0.030+
	(0.008)	(0.003)		(0.018)
Large firm	-0.001	-0.003*	-0.038***	0.008
	(0.003)	(0.001)	(0.003)	(0.006)
Tertiary education	0.048***	-0.018**	-0.005	0.033*
	(0.008)	(0.006)	(0.011)	(0.014)
Home owner	-0.210***	-0.004+	-0.002	0.002
	(0.004)	(0.002)	(0.004)	(0.007)

Age-squared	0.000*	-0.000	0.001***	-0.000+
	(0.000)	(0.000)	(0.000)	(0.000)
Age-cubed	0.000	0.000***	-0.000***	0.000***
	(0.000)	(0.000)	(0.000)	(0.000)
Constant	-0.225	-0.127	0.644**	0.823**
	(0.138)	(0.084)	(0.224)	(0.319)
Observations	231867	139738	141350	47041
Adjusted R^2	0.223	0.196	0.207	0.133

Note: Clustered standard errors in parentheses. + p < 0.1 * p < 0.05, ** p < 0.01, *** p < 0.001. Wave and region fixed-effects are included in all regressions. Satisfaction integer=0 is omitted and acts as the reference category.

B Additional Figures

Figure S1: The Predictive Power of a Feelings Integer: United Kingdom (cross-*sectional logit specification*)

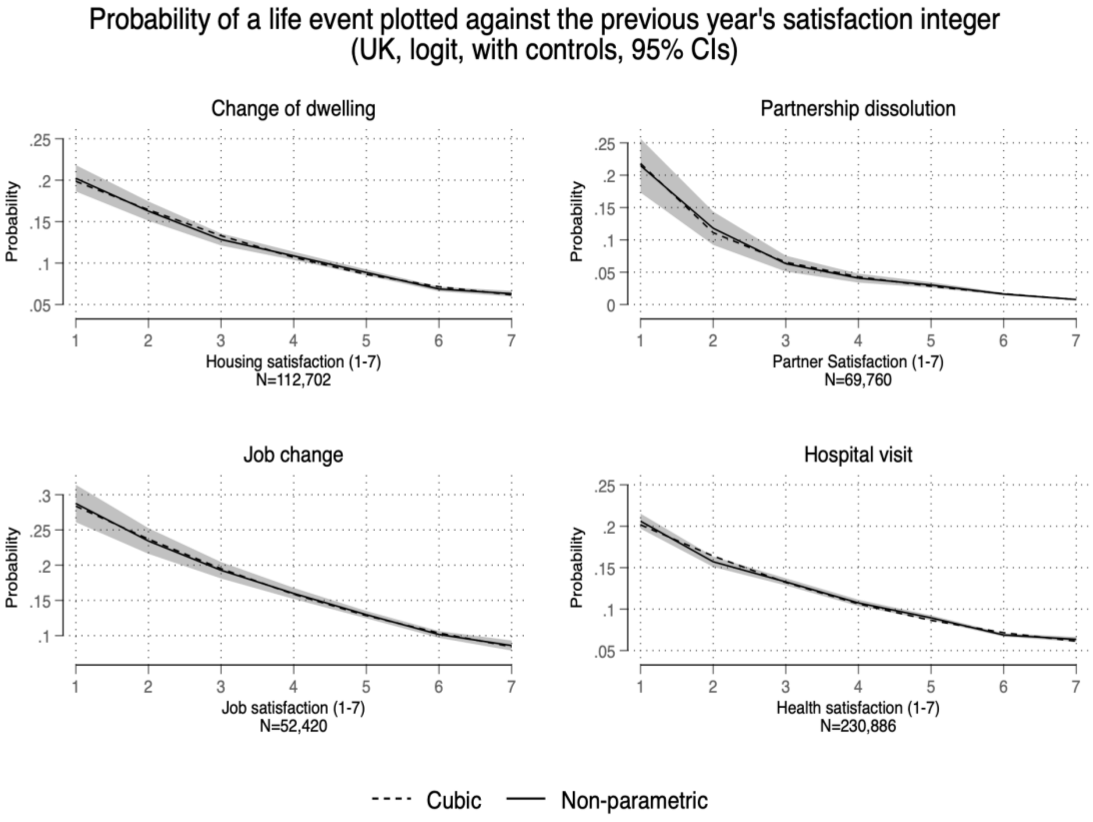

Figure S2 The Predictive Power of a Feelings Integer: Germany (cross-*sectional logit specification*)

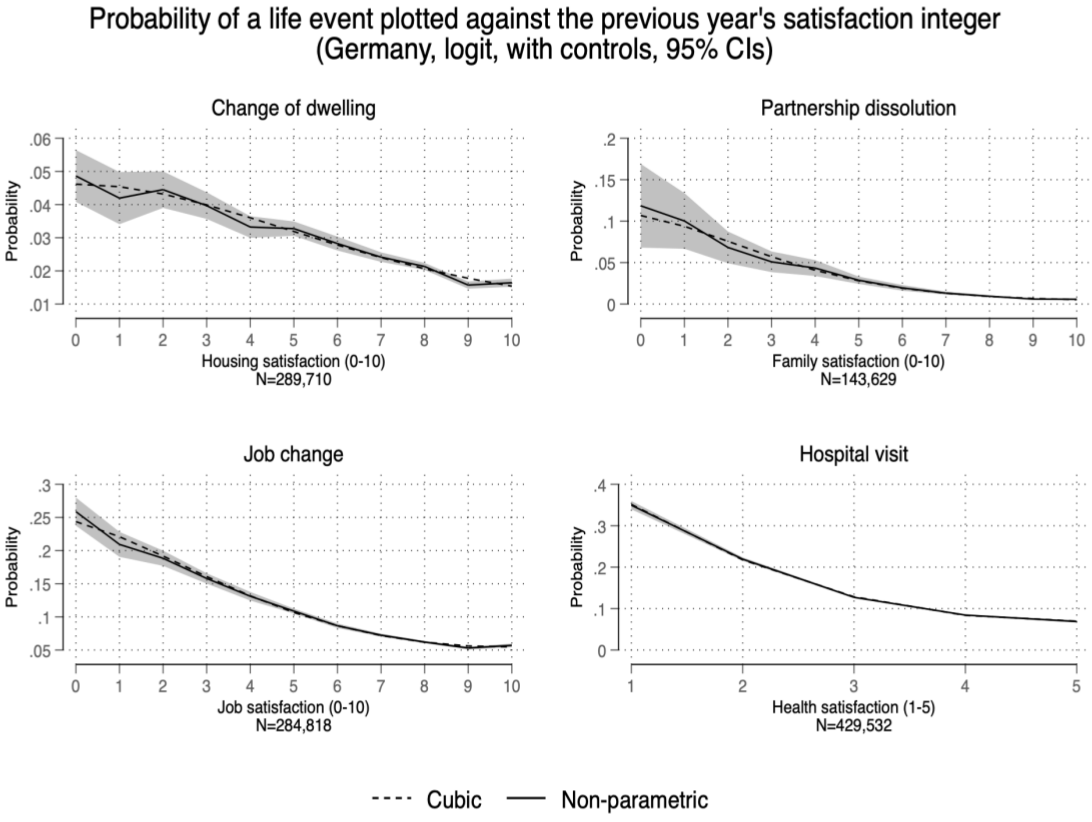

Figure S3 The Predictive Power of a Feelings Integer: Australia (cross-*sectional logit specification*)

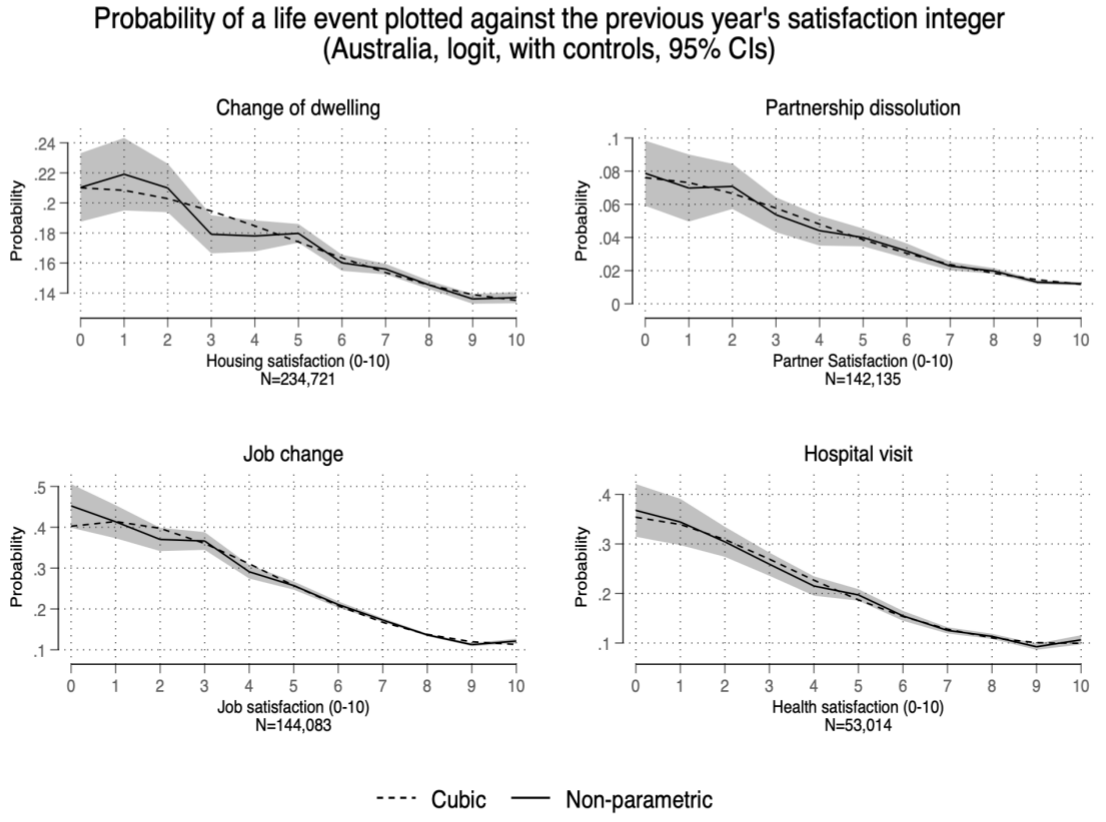

Supplement: Supplementary File [file pnas.2210412119.sapp.pdf]
